# Anatomical and positional variants of the brachiocephalic trunk in a Mexican population

**DOI:** 10.1186/s12880-021-00645-w

**Published:** 2021-08-14

**Authors:** Nadia Gabriela Jasso-Ramírez, Rodrigo E. Elizondo-Omaña, Ingrid Abigail Garza-Rico, Kouatzin Aguilar-Morales, Alejandro Quiroga-Garza, Guillermo Elizondo-Riojas, José Luis Treviño-González, Santos Guzman-Lopez

**Affiliations:** 1 Human Anatomy Department, School of Medicine, Universidad Autonoma de Nuevo Leon, Monterrey, Nuevo Leon Mexico; 2 Radiology and Imaging Department, Universidad Autonoma de Nuevo Leon, University Hospital “Dr. José Eleuterio González”, Monterrey, Nuevo Leon Mexico; 3 Instituto Mexicano del Seguro Social, Delegación de Nuevo Leon, Cirugia General, Monterrey, Nuevo Leon Mexico; 4 Otorhinolaryngology Department, Universidad Autonoma de Nuevo Leon, University Hospital “Dr. José Eleuterio González”, Monterrey, Nuevo Leon Mexico

**Keywords:** Brachiocephalic trunk, Morphology, Innominate artery, Tracheostomy, Major hemorrhage, Complications

## Abstract

**Background:**

Brachiocephalic trunk (BCT) variants may have a clinical impact during surgical procedures, some of which could be fatal. The objective of this study was to classify height positions of the BCT and report their prevalence in a Mexican population.

**Methods:**

Patients: A retrospective, descriptive, observational, and cross-sectional was performed using computed tomography angiography (CTA) of adult (> 18 years of age) patients, without gender distinction, of Mexican origin. Measuring techniques were standardized using the suprasternal notch to analyze linear and maximum heights, linear and curved lengths, and the vertebral origin and bifurcation levels of the BCT.

**Results:**

A total of 270 CTA were obtained (66.7% men and 33.3% women). A high position of BCT was present in 64.81% (n 175/270). The mean linear medial height was 0.58 ± 1.91 cm, the maximum height of the free edge was 3.85 ± 2.04 cm, side length of the midline at the maximum height of the free edge was 1.46 ± 2.59, linear length 3.72 ± 0.70, and a curve length 3.99 ± 0.79. The BCT origin was most predominant at the T3 (57.9%) and T4 (27.0%) vertebral levels, with the bifurcation at T2 (57.9%) and T1 (36.2%).

**Conclusions:**

There is a high prevalence of high position BCT in our population. Patients should be assessed before any procedures in the area, due to the potential risk of complications.

## Background

The brachiocephalic trunk (BCT) or innominate artery (IA) is a mediastinal artery that supplies blood to the arm, head, and neck on the right side of the body [1]. It originates along with the proximal portion of the subclavian artery from the fourth right aortic arch towards the end of the 5th week of embryological development [2–5]. The BCT is the first branch of the aortic arch, originating from its horizontal portion at the height of the upper edge of the second rib, anterior to trachea and right recurrent nerve, posterior to the brachiocephalic vein, its trajectory is cephalic and curved laterally to the right. At the sternoclavicular joint, the BCT bifurcates into its two terminal branches, the right common carotid, and the right subclavian artery. The left common carotid and left subclavian artery originate directly from the aortic arch [3, 6–12].

The BCT does not branch other arteries, although reports of the inferior thyroid, thymus, and bronchial branches have been described [11–14]. It is classified as a high-lying (prolonged) when if bifurcates cephalic to the sternoclavicular junction. This anatomical variant has been gradually recognized, reported by radiological studies with a prevalence of 3 to 26.4%, and has been correlated with potential surgical complications such as fistulas and bleeding in neck procedures, thyroid surgery, mediastinoscopy, percutaneous tracheotomy, subclavian catheters, among others [15–17].

The brachiocephalic artery has shown to be the most frequent eroded vessel [18], mostly due to pressure necrosis and mechanical abrasion from the tracheotomy tube. This can result in a tracheoinnominate fistula, which is fatal if not treated [1], or cause bleeding outside the trachea [17, 18]. An aberrant trunk can also compress the trachea triggering an airway obstruction syndrome [19]. It’s most common in the anterior middle wall [20, 21]. Surgical treatment is indicated when the symptoms are severe or multiple, do not respond to conservative treatment (management of comorbidities), and when the fibrobronchoscopy shows tracheal compression greater than 70%. The surgical procedure of choice is aortopexy [15, 22], which consists of the suture of the adventitia of the aorta to the sternum, performed with simultaneous evaluation of the tracheal lumen [16, 23, 24].

The embryological etiology of this anatomical variation is due to abnormal differentiation of the third pair of aortic arches, causing the artery to cross in front of the trachea after emerging from the aortic arch at a more distal or aberrant location [16, 21]. Although infrequent, it can be manifest as a pulsating mass in the anterior region of the neck, with fatal complications if not identified [15].

Previous studies have based classification using the tracheal rings, however, these are not reliable due to the variation in neck length between patients. For this reason, the suprasternal notch has been proposed as an anatomical landmark [17]. The risk of bias should also be considered, as many studies do not report inter- or intra-observer reliability coefficients for their measurements [17, 25].

Data are limited regarding the standardization for measurements, and a classification has not been established. The purpose of this study is to propose a measurement method that allows classifying the different possible anatomical positions that this artery may have.

## Material and methods

A retrospective, descriptive, observational, and cross-sectional study was performed. Computed tomography angiography (CTA) studies of head and neck were obtained from the database of the Radiology and Image Department of the University Hospital “Dr. José Eleuterio González.” Images from a 64-slices CTA (General Electric CT99 Light Speed VCT, Software 2978195VCT) were used. CTA parameters were: rotation 0.4 s helicoidal acquisition, 20 mm detector covering, 120 kV, with a window range of WW:600–650 and WL:170–225, 0.625 mm width slices, 0.53:1 mm/rot Pitch and 22 to 23 cm FOV.

CTAs from adult (> 18 years of age) patients, without gender distinction, of Mexican origin, were included for analysis. Those with a history of chest or neck surgeries, structural alterations, great tumors, artifacts, chest fractures, or other pathologies that modify the anatomy of the region, were excluded. Those with low image quality or unidentifiable arteries were eliminated.

The sample size was previously calculated based on the variability reported in the literature and a confidence of 95%. This resulted in a sample size of 263 brachiocephalic trunk arteries however, it was decided to expand the sample size to 270. Prior to the study, a subsample of 10 CTA scans were randomly selected, to standardize the measurement technique. Two observers (radiology head and neck experts) reviewed and analyzed the image studies. Studies were reviewed independently after establishing an interobserver variability kappa coefficient of 0.79 in a pilot sample.

**Fig. 1:**
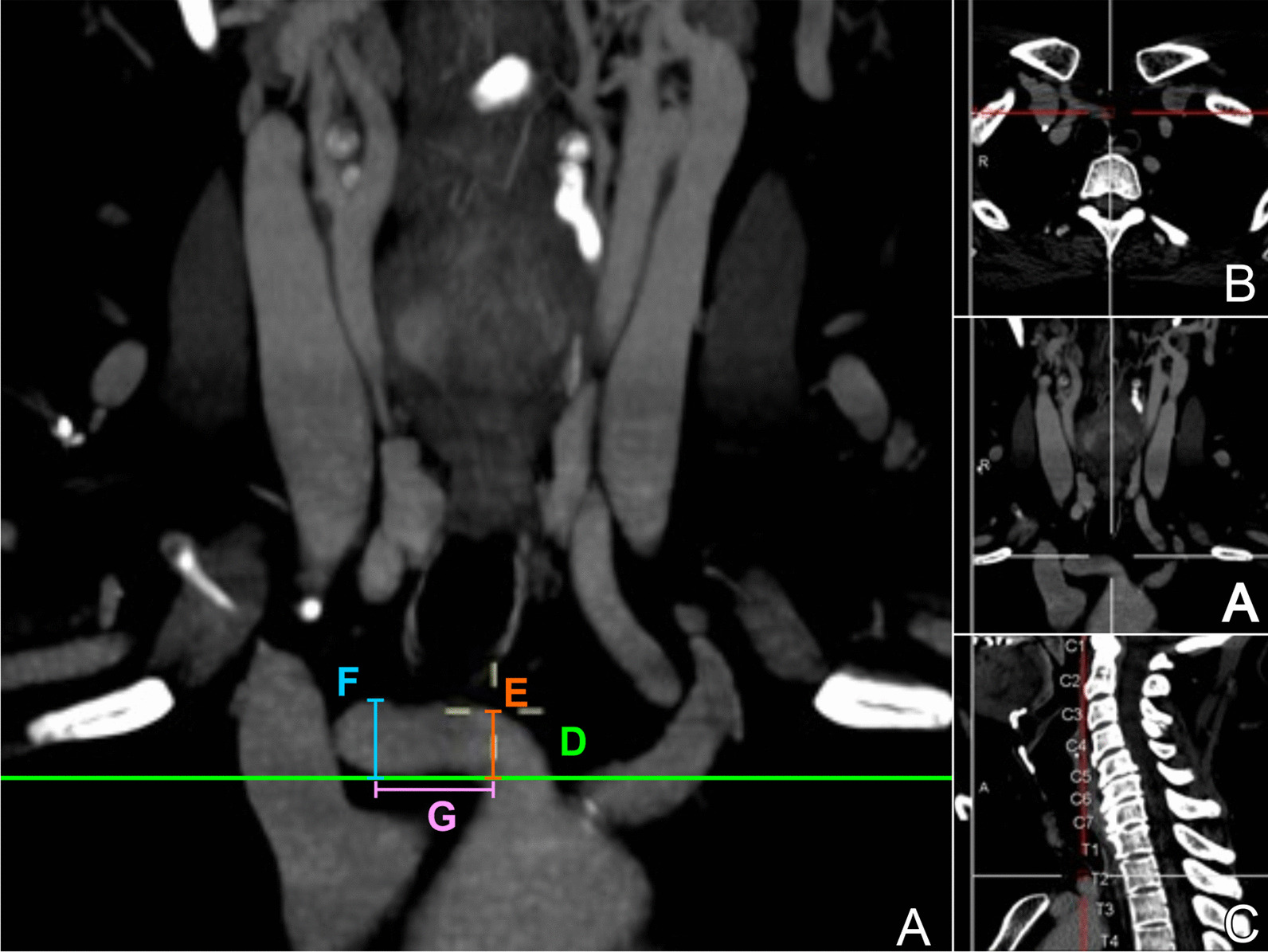
Neck computed tomography angiography (CTA) of the braquiochephalic trunk. **a**. Coronal view; **b** axial view; **c** sagittal view with vertebral level of origin of the trunk; (**d**/green) Suprasternal notch reference line; (**e**/orange) Linear medial height [LMH]; (**f**/blue) Maximum height at the free edge [MHFE]; (**g**/purple) Lateral length between MHFE and LMH

Measurement operations were performed with multiplanar reformatting using Centricity Universal Viewer. The studies were assessed separately by two expert head and neck radiologists, using a multiplanar reformatting of the tomography’s volume, the central point of the superior margin of the suprasternal notch was located, it allowed the ubication of all reference structures by the simultaneous visualization of sagittal, coronal and axial views that corresponded to the very same level the computer’s cursor was located (Fig. 1).

**Fig. 2:**
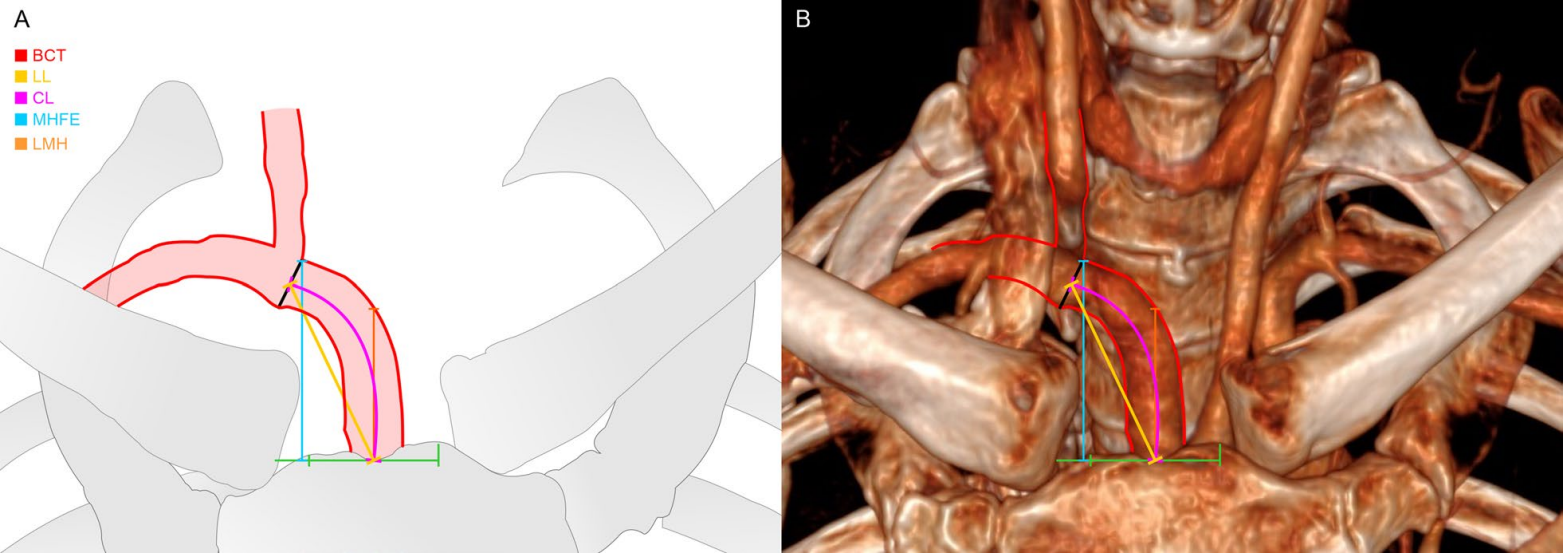
Measuremente of the braquiochephalic trunk. **a** Schematic diagram of measurements. **b** Anatomical reconstruction using computed tomography angiography (CTA), coronal view. BCT/red: braquiochephalic trunk; LL/yellow: linear length; CL/pink: curved length; MHFE/blue: maximum height at the free edge; LMH/orange: linear medial height; green line: suprasternal notch reference line **a** bifurcation of the braquiochephalic trunk (BCT); **b** curved length of the BCT; **c** linear medial height

For measurements, the scan was automatically centered and linear lengths were calculated by the software’s function “distance”, first the linear medial height (LMH) was measured according to the central y-axis, extending the cursor from the suprasternal notch to the midline highest point the BCT reached; for the highest position of the BCT before its bifurcation (maximum height at the free edge, MHFE), an x-axis was drawn touching this point, and then distance measure was made from the suprasternal midline point to the cross with the reference line. Distance between these drawn lines (LMH to MHFE) was also made (Figs. 1, 2).

Another function of this software allowed to measure the curve length of the artery before its bifurcation, demanding the drawing of the artery’s trajectory (Fig. 2).

The distance between the suprasternal notch and the cricoid and thyroid cartilages were measured according to the midline, from a suprasternal notch reference line to the inferior edge of each cartilage, although these distance measurements were not initially contemplated, therefore, were not statistically correlated with gender groups, however, the findings were deemed important to report.

Statistical analysis was performed using the SPSS Version 24.0 software for Windows 10. The mean and standard deviation for each measurement parameter were determined independently. The Mann Whitney U test was used to determine the significance of the differences between each measurement parameter between men vs women for each morphometric parameter, interpreting a value of p ≤ 0.05 as significant.

This study was previously reviewed and approved by the ethics and research committees Hospital Universitario “Dr. Jose Eleuterio Gonzalez” of the Universidad Autonoma de Nuevo Leon under the registration number AH18-00,009, certifying that it adheres to the guidelines of the General Health Law on Health Research in Human Beings of our country, as well as international guidelines and the Declaration of Helsinki. Due to the retrospective design of the study, informed consent was waived by the ethics and research committees Hospital Universitario “Dr. Jose Eleuterio Gonzalez” of the Universidad Autonoma de Nuevo Leon. None of the CTA were performed for the purposes of this study.

## Results

A total of 270 contrasted neck CTA were analyzed, with a mean age of 46.5 ± 14.6 years (men 45.8 ± 13.6 years, n 180 [66.7%], women 46.8 ± 15.1 years, n 90 [33.3%]). The brachiocephalic trunk was considered high-lying position when it exceeded the suprasternal notch without having bifurcated into its terminal branches; Table 1 represents our classification of the BCT reached heights. We report 22 cases (3F/19M) of a BCT bifurcated right at the level of the suprasternal notch. A BCT bifurcated above the suprasternal notch was found in 175 patients (64.81%) of the total population, being 70 cases of women (40%), and 105 cases of men (60%).

**Table 1:** Height classification

Distance	LMH n 270 (%)	MHFE n 270 (%)
Mean (cm ± SD)	0.58 ± 1.91	1.80 ± 2.05
< 1	169 (62.6)	64 (23.7)
1.0–1.5	48 (17.8)	55 (20.4)
1.6–2.0	26 (9.6)	57 (21.1)
2.1–3.0	25 (9.3)	75 (27.8)
3.1- 4.0	2 (0.7)	18 (6.7)
4.1–5.0	0 (0)	1 (0.4)

All values expressed in centimeters; *SD* standard deviation, *LMH* linear medial height, *MHFE* Maximum height at free edge

### Heights and lengths

Mean ± standard deviation was calculated for each parameter (Table 2). The mean LMH was 0.58 ± 1.91, MHFE of the BCT 1.80 ± 2.05, side length of the LMH at the MHFE 1.47 ± 1.13, Linear Length 3.72 ± 0.71, Curve Length 3.99 ± 0.79, Vertebral Origin Level 3.16 ± 0.73 and Vertebral Branch Level 1.83 ± 0.93 of the BCT in its subclavian artery and external carotid branches.

**Table 2:** BCT measurements

Variables	Total	Gender		P value
	Mean and SD	Females	Males	
Linear medial height (LMH)	0.58 ± 1.91	1.20 ± 2.76	0.27 ± 1.20	< 0.001*
Maximum height of the free edge (MHFE)	1.80 ± 2.05	2.20 ± 2.88	1.60 ± 1.43	0.003*
Lateral distance from LMH to MHFE	1.47 ± 1.13	1.31 ± 0.69	1.55 ± 1.29	0.034*
Lineal length	3.72 ± 0.71	3.48 ± 0.62	3.85 ± 0.72	< 0.001*
Curve length	3.99 ± 0.79	3.82 ± 0.73	4.08 ± 0.80	0.007*
Vertebral origin level	3 ± 1	3 ± 1	3 ± 1	< 0.001*
Vertebral bifurcation level	2 ± 1	2 ± 1	2 ± 1	0.105

All values are reported in centimeters (cm). Statistical test. * Significance set a < 0.05

Frequency of vertebral levels BCT’s position is reported in Table 3. The lowest position of the BCT was − 3.5 cm, and the highest 4.5 cm.

**Table 3:** Brachiocephalic trunk Vertebral level

Vertebral body	Origin (%)	Bifurcation (%)
C7	0.0	0.7
T1	0.7	36.2
T2	14.5	57.9
T3	57.9	5.3
T4	27.0	0.0

### Distance to cricoid and thyroid cartilages

The mean distances from the free edge of the BCT to the cricoid and thyroid cartilage were 2.02 cm and 3.22 cm respectively. In 64 (23.70%) studies the free edge was found less than 1 cm away from the cricoid cartilage, while in 5 patients the BCT exceeded this level by a mean of 8.8 mm, two of these reaching the thyroid cartilage.

## Discussion

A high position of BCT was present in 64.8% of the Mexican population. The bifurcation was most predominant at the vertebral level T2 (57.9%) and T1 (36.2%). The mean LMH was 0.58 ± 1.91 cm, with a MHFE of 3.85 ± 2.04 cm. This means there is a high prevalence of high-lying BCT, and will not be an unusual finding for surgeons during neck procedures.

Tracheostomy is a surgical procedure that dates back to 1500 BC, with the first references referring to the "wind tube" incisions described in the Ebers papyrus and the Rig Veda. Alexander the Great, Asclepiades, Aretaeus, and Galen are registered for having used this operation. From 1546 to 1883, thanks to Brassarolo's writings, the procedure was considered useless and irresponsible and few surgeons dared to do it [26].

In 1879, Kbrte [27] reported the first case of a tracheostomy complicated by the erosion of a major blood vessel. Schlaepfer in 1924 [28] reviewed 115 cases of tracheostomies performed and the outcome of which was fatal bleeding. The innominate artery ruptured in 83 patients, the carotid artery in five, the inferior thyroid artery in three, the superior thyroid artery in one, and the right innominate vein in four. Aortic aneurysms ruptured in two patients, and the source of the bleeding was unknown in 17. No other reports were made until 1956 when Davis and Southwick [29] reported two cases of rupture of the innominate.

For pre-surgical diagnosis and management, previous studies have used tracheal rings to assess the position of the BCT, locating the bifurcation between rings 6 and 13 [1] (Table 4). However, this method is inaccurate and insecure at the time of the evaluation of preoperative risks, in addition to not extrapolating the findings to other individuals since the length of each patient's neck is variable.

**Table 4:** BCT position literature review

Study	Method	Sample	Measurement reference	BCT positions	Maximum height	Minimum height
Comert et al. [15] Turkey	Cadaveric	1	Tracheal rings	Above 4th and 5th tracheal rings	–	–
Ozlugedik et al. [31] Turkey	MR angiography	1	Trachea	High, horizontal and anterior to the trachea	–	–
Gil-Carcedo et al. [1] Spain	Eco-doppler, CT angiography, MR angiography, surgical approach	2	Vertebral level	Cervical localization	–	–
Cai et al. [17] China	CT + surgical approach	829	Suprasternal notch	26.4% High laying BCTs Mean distance to BCT: 2.0 ± 0.4 cm	2.7 cm	0.8 cm
Weightman et al. [25] Australia	CT	500	Suprasternal notch	38.2% Bifurcated above suprasternal notch	–	–
Jasso-Ramirez (2021), Current study, Mexico	CT angiography	270	Suprasternal notch	Mean distance to BCT: 1.80 ± 2.05 cm 64.81% Bifurcated above suprasternal notch	4.5 cm	− 3.5 cm

Cai et al. [17] proposed using the suprasternal notch as a measurement parameter. This provided a reliable clinical and radiological anatomical reference, constant in relation to the patient's position. For this study, a high position BCT was established in all those who exceeded the suprasternal notch (64.8%, n 175/270). A sample of 829 reported a prevalence of 26.4% (n 219/829) for BCTs positioned above the suprasternal recess, and reported 2.2% (n 18/829) with a height that exceeded 2 cm. Our study includes the vertebral level of bifurcation, although this cannot be extrapolated to all age groups due to aging changes and shortening of vertebral body height due to fractures, osteoporosis, among others [30].

Cai et al. [17] also reported a mean MHFE of 2.0 ± 0.4 cm, comparable to ours (1.80 ± 2.05 cm). However, they report a BCT position range of 2.7–0.8 cm in relation to the sternal notch, while our results included ranges between 3.10 and − 3.50 cm at the midline, and 4.50 and − 1.60 cm MHFE. Weightman and Gibbs [25] examined 500 CT of adult patients, identifying 53% (n 264) has a large vessel (brachiocephalic artery, right carotid artery, left carotid artery and unnamed left and right veins) anterior to the trachea in the anterior triangle of the neck. The most common was the BCT (38.2%, n 191) bifurcating above the suprasternal notch, but none of them reached the cricothyroid membrane. An important difference is noted between 26.4% prevalence in Chinese, 38.2% in Australian and 34.8% in Mexican populations, suggesting an ethnical influence (Table 4). More studies from different geographical areas are needed to establish a pattern.

Ozlugedik et al., reported only 2 publications that describe an IA that reaches the cricoid [31]. Faggioli et al. [32] describe the high bifurcation of IA as very rare, with no more than 5 cases at the height of the thyroid gland. These studies justified adding this measurement to our study, finding that the BCT exceeded the level of the cricoid cartilage in 5 patients, of which 2 patients presented BCT anterior to the cricothyroid membrane reaching even the thyroid cartilage. This low frequency can support the safety of cricothyroidotomy on tracheotomy in emergency access to the respiratory tract, however, we emphasize the importance of accurately identifying the cricothyroid membrane and a careful dissection by the surgeon, as well as the use of imaging studies, when available to properly assess the anatomy [33, 34].

There are reports regarding the anatomical variability of the main structures of the neck, measurements, distances between them and relationship with the vertebral bodies [35], however our study is the first to assess the position of the BCT from several angles, measuring its own length and distances from this to other important reference structures during a surgical approach (suprasternal notch, cricoid and thyroid cartilage). Several authors have described the anatomical vascular variants that may involve a surgical risk, however few make reference to the BCT and its variants, and most are only case reports. Our findings in addition to those of Cai et al., and Weightman and Gibbs [17, 25] suggest that it is important to recognize that these abnormalities in the origin and bifurcation of BCT are much more frequent than previously reported.

### Limitations

Due to the retrospective design of our study, the position of the patients' heads was not standardized during the taking of the CTA, therefore we declare that this may have influenced the distances measured from the BCT to the cricoid and thyroid cartilages.

## Conclusion

After assessing the different heights of presentation of BCT and its relationship with neck structures, we recognize a high and risky position of this arterial segment is not so infrequent. The BCT has been poorly described, and surgeons should consider a high position of this artery during emergency neck procedures, and preoperatively indicate imaging studies to evaluate patient safety.

## Data Availability

The datasets generated and/or analyzed during the current study are not publicly available as these are in Spanish with the use of internal codifications and may include personal registration data from the studies which would need to be edited out, but are available from the corresponding author on reasonable request.

## Abbreviations

BCT: Brachiocephalic trunk
CTA: Computed tomography angiography
IA: Innominate artery
LMH: Linear medial height
MHFE: Maximum height at the free edge

## Declarations

**Ethical approval and consent to participate**

This study was previously reviewed and approved by the ethics and research committees Hospital Universitario “Dr. Jose Eleuterio Gonzalez” of the Universidad Autonoma de Nuevo Leon under the registration number AH18-00009, certifying that it adheres to the guidelines of the General Health Law on Health Research in Human Beings of our country, as well as international guidelines and the Declaration of Helsinki. Due to the retrospective design of the study, informed consent was waived by the ethics and research committees Hospital Universitario “Dr. Jose Eleuterio Gonzalez” of the Universidad Autonoma de Nuevo Leon. None of the CTA were performed for the purposes of this study.
